# Discrimination and Adverse Perinatal Health Outcomes: A Latent Class Analysis

**DOI:** 10.5888/pcd20.230094

**Published:** 2023-11-02

**Authors:** Emily A. Doherty, Kathleen Cartmell, Sarah Griffin, Moonseong Heo, Liwei Chen, Jessica L. Britt, Amy H. Crockett

**Affiliations:** 1 Department of Public Health Sciences, College of Behavioral, Social and Health Sciences, Clemson University, Clemson, South Carolina; 2 Department of Epidemiology, Fielding School of Public Health, University of California, Los Angeles; 3 Division of Maternal-Fetal Medicine, Department of Obstetrics and Gynecology, Prisma Health, Greenville, South Carolina; 4 University of South Carolina School of Medicine, Greenville, South Carolina

## Abstract

**Introduction:**

An intersectionality framework recognizes individuals as simultaneously inhabiting multiple intersecting social identities embedded within systems of disadvantage and privilege. Previous research links perceived discrimination with worsened health outcomes yet is limited by a focus on racial discrimination in isolation. We applied an intersectional approach to the study of discrimination to examine the association with adverse perinatal health outcomes.

**Methods:**

We analyzed data from a cohort of 2,286 pregnant participants (Black, n = 933; Hispanic, n = 471; White, n = 853; and Other, n = 29) from the Centering and Racial Disparities trial. Perceived discrimination was assessed via the Everyday Discrimination Scale (EDS) and perinatal health outcomes collected via electronic medical record review. Latent class analysis was used to identify subgroups of discrimination based on EDS item response and the rate of adverse perinatal health outcomes compared between subgroups using a Bolck, Croon and Hagenaars 3-step approach.

**Results:**

Four discrimination subgroups were identified: no discrimination, general discrimination, discrimination attributed to one or several social identities, and discrimination attributed to most or all social identities. Experiencing general discrimination was associated with postpartum depression symptoms when compared with experiencing no discrimination among Black (9% vs 5%, *P = .*04) and White participants (18% vs 9%, *P = .*01). White participants experiencing general discrimination gave birth to low birthweight infants at a higher rate than those experiencing no discrimination (11% vs 6%, *P = .*04). No significant subgroup differences were observed among Hispanic participants.

**Conclusion:**

Perceived discrimination may play an influential role in shaping perinatal health. More research applying an intersectional lens to the study of discrimination and perinatal health outcomes is needed.

**Summary**

**What is known on this topic?**

Discrimination is consistently associated with poor health outcomes and health disparities, including for perinatal health, yet few studies address intersectional discrimination.

**What is added by this report?**

We evaluated discrimination based on multiple social identities and assessed differential associations with adverse perinatal health outcomes. We found that Black and White participants exposed to general discrimination were more likely to experience symptoms of postpartum depression and that White participants delivered more low birthweight infants relative to those who experienced no discrimination.

**What are the implications for public health practice?**

Perceived discrimination in pregnancy can be associated with adverse perinatal health outcomes. Addressing intersectional discrimination exposure may promote perinatal health.

## Introduction

Racial and ethnic disparities in perinatal health are present across many countries but are particularly pronounced in the US. Infants of Black pregnant people die at more than twice the rate of those of White people, and Black pregnant people themselves are 3 times more likely to die during pregnancy (1,2). Perinatal health disparities are also seen by health insurance status, age, and weight (2–4). One explanation for the origin of these disparities is the increased burden of stress associated with exposure to persistent discrimination experienced over the life course. Discrimination is thought to affect health through dysregulation of psychological and physiological stress responses systems (eg, altered hypothalamic-pituitary-adrenal axis activation; elevated blood pressure, heart rate, and cortisol production; and inflammation) and accelerated aging, as well as through altered engagement in health behaviors (increased participation in unhealthy behaviors and nonparticipation in healthy behaviors) (5,6). A large and growing body of research demonstrates the negative effect of perceived discrimination on health (7) and suggests that discrimination is a risk factor for adverse perinatal health outcomes (APHOs) including preterm birth (PTB), low birthweight (LBW), small for gestational age, and hypertensive disorders of pregnancy (8).

Existing literature assessing the impact of discrimination on health has been limited by a focus on discrimination based on a single dimension, most commonly race-based discrimination (9). A focus exclusively on racial discrimination may mask complexities in the maternal discrimination experience and potentially underestimates the overall impact of discrimination on perinatal health (10). Adopting an intersectionality framework recognizes that individuals simultaneously occupy multiple interconnected social identities (eg, race, ethnicity, gender, sexual orientation, socioeconomic status) that confer privilege or disadvantage (11,12). Latent class analysis (LCA) offers one method to apply an intersectional approach in quantitative analysis (13). LCA is a data-driven method that probabilistically assigns individuals to latent subgroups based on observed categorical indicator variables (14).

In this study, we aimed to 1) classify mutually exclusive subgroups of pregnant people based on patterns of response to Everyday Discrimination Scale (EDS) items through LCA and 2) examine whether subgroups characterizing different patterns of discrimination were differentially associated with APHOs.

## Methods

### Participants, design, and setting

We analyzed data from the Centering and Racial Disparities (CRADLE) study (ClinicalTrails.gov identifier no. NCT02640638), a randomized controlled trial of pregnant people (N = 2,348) conducted at a single obstetrics and gynecology practice in Greenville, South Carolina. The primary objective of the CRADLE study was to compare the rate of PTB and LBW of patients who participated in group prenatal care (GPNC, a novel model of prenatal care combining clinical assessment, prenatal education, and peer socialization) with their counterparts in standard individual prenatal care (IPNC), as well as racial disparities in these outcomes. The CRADLE study was approved by the Prisma Health institutional review board (no. Pro00043994). The full study protocol and primary findings have been published previously (15,16).

The study population was medically low-risk pregnant people of diverse races and ethnicities. Eligible patients were aged between 14 and 45 years, were less than 24 weeks gestational age at enrollment, and were proficient in English or Spanish. Exclusion criteria were medical or pregnancy complications that would preclude prenatal care and delivery by a nurse practitioner or nurse midwife (ie, pregestational diabetes, chronic hypertension requiring medication, any disease requiring immunosuppression, a body mass index of more than 50 kg/m^2^, multiple gestation, patients anticipating a planned preterm delivery or planned cerclage, or lethal fetal anomalies) or patients with medical, social, or behavioral conditions that would preclude participation in group care (ie, active pulmonary tuberculosis, current incarceration, or severe uncontrolled psychiatric illness). In the CRADLE study, participants were randomly allocated 1:1 stratified by race and ethnicity to attend GPNC or IPNC. Trial intervention and control groups were combined and included in our analysis.

### Data collection

Study recruitment took place between February 2016 and March 2020. Participants were followed from enrollment through delivery and 12 weeks postpartum. Data were collected at 3 points: 1) an initial survey at the baseline visit between 8 and 23 weeks gestational age, 2) a second survey between 30 and 40 weeks gestational age, and 3) a medical chart abstraction 12 weeks postpartum. Surveys included demographic questions and various psychosocial and behavioral measures. Medical and delivery information were collected through manual chart abstraction as well as automated query of the electronic medical record (EPIC Systems Inc).

### Measures

Indicator variables used to define unobserved latent class membership comprised patient response to the adapted 11-item Everyday Discrimination Scale (EDS) administered at baseline (17). The EDS is among the most commonly used measures of discrimination and has high reliability and construct validity (8). The EDS attempts to measure chronic but minor instances of discrimination. It first asks respondents about their day-to-day experience of 10 forms of unfair treatment. Response values are on a 4-point Likert scale ranging from “never” to “often.” Respondents who indicate any discrimination are then asked to identify the reasons for their mistreatment and can select multiple reasons including those related to gender, race and ethnicity, insurance and Medicaid status, ancestry and national origin, age, religion, weight or some other aspect of physical appearance, sexual orientation, and education or income level. We formed a binary variable of discrimination frequency consisting of “never” versus “rarely, sometimes, or often.” Each attribution for discrimination was coded as a binary variable with possible responses of either yes or no; attributions with low prevalence were combined to form an “other” discrimination variable.

The primary outcome was a composite measure of APHOs. A binary variable was created representing indication of none versus 1 or more of the following 7 outcomes: PTB (delivery at <37 weeks gestation); LBW (infant birthweight <2,500 g); small for gestational age (SGA, birthweight below the 10th percentile for gestational age); infant admission to the neonatal intensive care unit (NICU); 5-minute Apgar score <7; pre-eclampsia; and patient admission to the intensive care unit (ICU). Individual APHO’s composite components, as well as postpartum depression symptoms (PPDS), were considered as secondary outcomes. PPDS was identified based on Edinburgh Postnatal Depression Scale (EPDS) response (18). The EPDS is a widely used 10-item screening instrument for depression risk, which has high sensitivity and specificity in detecting depressive disorders with a cutoff of 13 (19). The EPDS was routinely administered at the postpartum outpatient visit as part of routine clinical care and the results abstracted from the medical record at 12 weeks postpartum; we used a binary PPDS variable (scores <13 vs ≥13).

Self-reported sociodemographic characteristics were collected through the baseline survey and included race and ethnicity (Black, Hispanic, White, or other); age (14–24 y, 25–34 y, and 35–45 y); Medicaid eligible (yes or no); educational attainment (less than high school, high school degree, more than high school degree); current relationship with baby’s father (categorized as married, engaged, or in a committed dating relationship, or single or other relationship); nativity (born in the US vs born outside the US); parity (nulliparous vs primiparous or multiparous); and body mass index (BMI) at initial prenatal care visit (underweight, <18.5 kg/m^2^; healthy weight 18.5 kg/m^2^ to <25.0 kg/m^2^; overweight 25.0 kg/m^2^ to <30.0 kg/m^2^; or obese, ≥30.0 kg/m^2^). Participants identified their race and ethnicity through questions used by the US Census Bureau, which allowed participants to select multiple categories, as well as providing a space for open-ended description of race and ethnicity (20).

### Statistical analyses

All statistical analyses were performed by using SAS version 9.4 (SAS Institute, Inc). First, sample characteristics were described and differences by race and ethnicity were examined by using χ^2^ tests. LCA models were then estimated by using SAS PROC LCA and the LCA Bootstrap Macro (21,22). To identify an optimal LCA model, models with between 1 and 6 latent classes were tested. Optimal models were indicated by minimum Akaike information criterion (AIC) and Bayesian information criterion (BIC) values in addition to the Bootstrap Likelihood Ratio Test (BLRT) that compares model fit for k classes relative to k+1 classes. Two primary sets of parameters were estimated: class membership probabilities (the size of the latent class identified) and item response probabilities (the conditional probability of a response given class membership). Item response probabilities were used to label latent classes. A likelihood ratio difference test was used to test equality across race and ethnicity following a 3-step approach, and race and ethnicity groups were modeled separately (14).

The Bolck, Croon and Hagenaars (BCH) 3-step approach was used to assess whether latent classes were associated with APHOs, applied separately for each outcome (23). Parameters of the LCA model were first estimated without distal outcomes, posterior probabilities of latent class membership were then used to compute a weighting variable, and the association between the weighted variable and the distal outcome were investigated using logistic regression. The %LCA_Distal_BCH macro provides an overall test of association between class membership and outcomes of interest, as well as pairwise comparisons of the expected values between classes using Wald tests (23). A *P* value of <.05 was considered significant.

A sensitivity analysis using maximum-probability assignment was performed. Multiple logistic regressions were conducted to test whether prenatal care assignment in the CRADLE study modifies the link between latent classes and APHOs.

## Results

### Descriptive statistics

Of the 2,348 CRADLE study participants, 2.6% (n = 62) participants were excluded due to missing values on all indicator variables, resulting in a final analytic sample of 2,286. More than 40% of the sample identified as Black, 20.6% as Hispanic, 37.3% as White, and 1.3% as other race and ethnicity (Table 1). Most participants were aged 25 to 34 years (76.8%), Medicaid eligible (96.4%), had a high school education (53.6%), were engaged or in a committed relationship with the baby’s father (39.9%), had previously given birth (55.5%), were born in the US (83.9%), and were overweight or obese (64.3%). The frequency of these sociodemographic characteristics significantly differed across racial and ethnic groups (*P < .*001).

**Table 1:** Sociodemographic Characteristics, Everyday Discrimination, and Adverse Perinatal Health Outcomes of Participants of the Centering and Racial Disparities Study (N = 2,286)

Characteristic/variable	Overall	Black	Hispanic	White	*P* value^a^
	Frequency (%)				
**Sociodemographic characteristics**					
**Race and ethnicity**					
Black	933 (40.8)	—	—	—	NA
Hispanic	471 (20.6)	—	—	—	
White	853 (37.3)	—	—	—	
Other	29 (1.3)	—	—	—	
**Age, y**					
14–24	401 (17.5)	155 (16.6)	74 (15.7)	170 (19.9)	<.001
25–34	1,755 (76.8)	740 (79.3)	343 (72.8)	649 (76.1)	
35–45	130 (5.7)	38 (4.1)	54 (11.5)	34 (3.9)	
**Medicaid eligibility**					
Eligible	1,970 (96.4)	795 (96.9)	417 (96.1)	734 (96.2)	.63
Ineligible	73 (3.6)	25 (3.1)	17 (3.9)	29 (3.8)	
**Educational attainment**					
Less than high school	556 (24.3)	150 (16.1)	169 (35.9)	232 (27.2)	<.001
High school degree	1,226 (53.6)	562 (60.2)	202 (42.9)	446 (52.3)	
More than high school degree	460 (20.1)	203 (21.8)	87 (18.5)	162 (18.9)	
Missing	44 (1.9)	18 (1.9)	13 (2.8)	13 (1.5)	
**Relationship status**					
Married	422 (18.5)	261 (27.9)	52 (11.0)	137 (16.1)	<.001
Engaged or committed dating relationship with the baby’s father	913 (39.9)	59 (6.3)	171 (36.3)	180 (21.1)	
Single or other	451 (19.7)	374 (40.1)	176 (37.4)	352 (41.3)	
Missing	500 (21.9)	239 (25.6)	72 (15.3)	184 (21.6)	
**Nativity**					
Born outside the US	358 (15.7)	23 (2.5)	309 (65.6)	13 (1.5)	<.001
Born in the US	1,917 (83.9)	910 (97.5)	154 (32.7)	838 (98.2)	
Missing	11 (0.5)	0 (0)	8 (1.7)	2 (0.2)	
**Initial body mass index, kg/m^2^**					
Underweight (<18.5)	72 (3.1)	36 (3.9)	8 (1.7)	28 (3.3)	<.001
Healthy weight (18.5 to <25.0)	744 (32.6)	300 (32.2)	136 (28.9)	301 (35.3)	
Overweight (25.0 to <30.0)	577 (25.2)	206 (22.1)	155 (32.9)	207 (24.3)	
Obese (≥30.0)	893 (39.1)	391 (41.9)	172 (36.5)	317 (37.2)	
**Parity**					
Nulliparous	1,018 (44.5)	445 (47.7)	170 (36.1)	391 (45.8)	<.001
Primiparous or multiparous	1,268 (55.5)	488 (52.3)	301 (63.9)	462 (54.2)	
**Indicator variables**					
**Frequency of discrimination**					
Never	1,117 (48.9)	452 (48.5)	270 (57.3)	384 (45.0)	<.001
Rarely, sometimes, or often	1,169 (51.1)	481 (51.6)	201 (42.7)	469 (54.9)	
**Discrimination attribution**					
Age	344 (15.1)	156 (16.7)	27 (5.7)	157 (18.4)	<.001
Race and ethnicity	325 (14.2)	189 (20.3)	89 (18.9)	37 (4.3)	<.001
Weight or some other aspect of physical appearance	266 (11.6)	113 (12.1)	27 (5.7)	120 (14.1)	<.001
Gender	259 (11.3)	146 (15.7)	28 (5.9)	80 (9.4)	<.001
Education or income level	228 (9.9)	98 (10.5)	26 (5.5)	102 (11.9)	<.001
Other^b^	148 (6.5)	65 (6.9)	31 (6.6)	47 (5.5)	.44
**Outcome variables**					
**Composite adverse perinatal health outcomes**					
None	1,555 (68.0)	576 (61.7)	354 (75.2)	601 (70.5)	<.001
Any	731 (31.9)	357 (38.3)	117 (24.8)	252 (29.5)	
**Preterm birth (delivery at <37 weeks gestation)**					
No	1,954 (90.5)	784 (89.2)	412 (92.2)	732 (90.7)	.20
Yes	205 (9.5)	95 (10.8)	35 (7.8)	75 (9.3)	
Missing	127	54	24	46	
**Low birthweight (infant birthweight <2,500 g)**					
No	1,828 (90.8)	702 (87.6)	410 (93.6)	691 (92.3)	<.001
Yes	186 (9.2)	99 (12.4)	28 (6.4)	58 (7.7)	
Missing	272	132	33	104	
**Small for gestational age (birthweight below the 10th percentile for gestational age)**					
No	1,660 (82.5)	602 (75.3)	394 (89.5)	644 (85.9)	<.001
Yes	353 (17.5)	198 (24.8)	46 (10.5)	105 (14.0)	
Missing	273	133	33	104	
**Neonatal intensive care unit admission**					
No	1,848 (97.9)	738 (97.9)	402 (99.3)	683 (97.3)	.08
Yes	38 (2.0)	16 (2.1)	3 (0.7)	19 (2.7)	
Missing	400	179	66	151	
**Apgar score <7**					
No	2,051 (93.3)	818 (91.6)	436 (95.2)	772 (93.9)	.03
Yes	148 (6.7)	75 (8.4)	22 (4.8)	50 (6.1)	
Missing	87	40	13	31	
**Preeclampsia**					
No	2,117 (92.6)	856 (91.8)	442 (93.8)	791 (92.7)	.36
Yes	169 (7.4)	77 (8.3)	29 (6.2)	62 (7.3)	
**Intensive care unit**					
No	2,280 (99.7)	931 (99.8)	470 (99.8)	850 (99.7)	.83
Yes	6 (0.3)	2 (0.2)	1 (0.2)	3 (0.4)	
**Postpartum depression**					
Score <13	1,413 (90.2)	586 (92.3)	324 (94.2)	486 (85.6)	<.001
Score ≥13	154 (9.8)	49 (7.7)	20 (5.8)	82 (14.4)	
Missing	719	298	127	285	

Abbreviation: NA, not applicable.

a *P* values determined by using χ^2^ test.

b Other discrimination includes discrimination attributed to insurance or Medicaid status, ancestry or national origin, sexual orientation, or religion.

### Measures of discrimination

Half of participants (51.1%) reported experiencing discrimination rarely, sometimes, or often (Table 1). Many participants attributed discrimination to age (15.1%), followed by race and ethnicity (14.2%), weight or some aspect of physical appearance (11.6%), gender (11.3%), education or income (9.9%), and other characteristics (6.5%). Apart from attribution to the combined “other” characteristic variable, participant reports of discrimination significantly differed by race and ethnicity (*P < .*001). Fewer Hispanic participants (42.7%) reported experience of discrimination relative to Black (51.6%) and White (54.9%) participants. White participants were least likely to attribute discrimination to race or ethnicity (4.3%) and to any “other” characteristic (5.5%), while Hispanic participants were least likely to attribute discrimination to all other factors (5.5%–5.9%).

Overall, 31.9% of the sample had an APHO (Table 1). Black participants had a higher rate of APHOs (38.3%, n = 357) relative to Hispanic (24.8%, n = 117) and White (29.5%, n = 252) participants. Prevalence of individual outcomes ranged from less than 1% for ICU admission to 17.5% for small for gestational age. White participants (14.4%) had higher rates of PPDS than Black (7.7%) and Hispanic (5.8%) participants. Due to collection at the postpartum visit, missingness on the PPDS variable was considerably higher than for other outcome variables (719 [31.5%] participants).

### Latent class models

Fit indices for models ranging from 1 to 6 classes are presented in Table 2. Classes 1–4 were well identified (higher % of seeds associated). Entropy for models ranged between 1.00 and 0.80, suggesting low classification uncertainty. The BIC suggests a 3-class model, while the AIC suggests that a 4-class model offers the best fit. The 4-class model was supported by the BLRT and yielded interpretable and meaningful classes; it was therefore selected to offer the best fit. Table 3 displays the latent class profiles and labels for the 4-class model. 

**Table 2:** Fit Indices for Latent Classes of Maternal Discrimination in the Overall Sample and Among Black, Hispanic, and White Participants, Centering and Racial Disparities Study^a^

	Model	G^2^	*df*	AIC	BIC	CAIC	SABIC	BLRT	Entropy	% of Seeds associated
Overall	1-class	2,724.45	120	2,738.45	2,778.60	2,785.60	2,756.36	NA	1.00	100
	2-class	308.26	112	338.26	424.28	439.28	376.62	0.01	0.85	100
	3-class	131.43	104	177.43	309.33	332.33	263.25	0.01	0.88	99.0
	4-class	**84.55**	**96**	**146.55**	**324.32**	**355.32**	**225.82**	**0.01**	**0.85**	**34.0**
	5-class	60.81	88	138.81	362.46	401.46	238.55	0.02	0.88	3.7
	6-class	45.02	80	139.02	408.55	455.55	259.22	0.10	0.80	5.4
Black	1-class	1,407.69	120	1,407.69	1,421.69	1,462.56	1,433.33	NA	1.00	100
	2-class	201.49	112	231.49	231.49	304.06	319.06	0.01	0.89	100
	3-class	89.58	104	135.58	269.86	269.86	173.81	0.01	0.87	100
	4-class	**60.57**	**96**	**122.57**	**272.56**	**303.56**	**174.11**	**0.01**	**0.90**	**34.7**
	5-class	45.42	88	123.42	312.12	351.12	188.25	0.12	0.92	46.7
	6-class	38.08	80	132.08	359.49	406.49	210.22	0.79	0.89	3.5
Hispanic	1-class	514.03	120	514.03	528.03	557.11	564.11	NA	1.00	100
	2-class	78.89	112	108.89	171.21	186.21	123.61	0.01	0.86	100
	3-class	50.70	104	96.70	192.26	215.26	119.26	0.01	0.93	96.6
	4-class	**33.51**	**96**	**95.51**	**224.31**	**255.31**	**125.92**	**0.04**	**0.89**	**50.4**
	5-class	22.39	88	100.39	262.43	301.43	138.65	0.19	0.91	11.3
	6-class	16.17	80	110.17	305.45	352.45	156.28	0.62	0.86	13.7
White	1-class	881.57	120	895.57	928.81	935.81	906.58	NA	1.00	100
	2-class	123.48	112	153.48	224.71	239.71	177.07	0.01	0.81	100
	3-class	69.32	104	115.32	224.54	247.54	151.50	0.01	0.83	100
	4-class	**50.73**	**96**	**112.73**	**259.94**	**290.94**	**161.49**	**0.06**	**0.86**	**44.9**
	5-class	39.63	88	117.63	302.83	341.83	178.98	0.33	0.84	61.5
	6-class	34.56	80	128.56	351.75	398.75	202.49	0.98	0.87	0.3

Abbreviations: AIC, Akaike information criterion; BIC, Bayesian information criterion; BLRT, bootstrap likelihood ratio test; CAIC, consistent AIC; G^2^, goodness of fit test; NA, not applicable; SABIC, sample size–adjusted BIC.

a Bolded numbers indicate the best-fitting models. A likelihood-ratio difference test (free: G^2^ = 145.96, *df* = 290; constrained: G^2^ = 246.66, *df* = 346; ∆G^2^ = 100.7, *df* = 56, *P < .*00) indicated that measurement invariance should be rejected.

**Table 3:** Item-Response Probabilities for 4-Class Models of Maternal Discrimination, Centering and Racial Disparities Study

Indicator items	Item response probabilities			
Overall	Class 1: no discrimination (49.1%)	Class 2: general discrimination (32.3%)	Class 3: education and income discrimination (8.8%)	Class 4: gender, race and ethnicity, and age discrimination (9.8%)
Discrimination frequency	0.00	0.99	0.99	0.99
Gender	0.00	0.09	0.00	0.83
Race and ethnicity	0.00	0.21	0.11	0.64
Age	0.00	0.17	0.31	0.67
Education and income	0.00	0.02	0.66	0.34
Weight and appearance	0.00	0.16	0.33	0.37
Other discrimination^a^	0.00	0.05	0.19	0.31
**Black**	**Class 1: no discrimination (48.9%)**	**Class 2: general discrimination (32.9%)**	**Class 3: gender, race and ethnicity, and age discrimination (12.5%)**	**Class 4: compound discrimination (5.6%)**
Discrimination frequency	0.01	0.99	0.99	0.99
Gender	0.00	0.00	0.91	0.71
Race and ethnicity	0.00	0.24	0.61	0.82
Age	0.00	0.17	0.54	0.79
Education and income	0.00	0.17	0.07	0.69
Weight and appearance	0.00	0.20	0.21	0.50
Other discrimination^a^	0.00	0.08	0.12	0.49
**Hispanic**	**Class 1: no discrimination (59.2%)**	**Class 2: general discrimination (31.0%)**	**Class 3: other discrimination** **(6.1%)**	**Class 4: compound discrimination (3.6%)**
Discrimination frequency	0.03	0.99	0.99	0.99
Gender	0.00	0.09	0.00	0.78
Race and ethnicity	0.00	0.45	0.22	0.91
Age	0.00	0.11	0.09	0.41
Education and income	0.00	0.06	0.26	0.54
Weight and appearance	0.00	0.14	0.00	0.34
Other discrimination^a^	0.00	0.00	0.76	0.55
**White**	**Class 1: no discrimination (45.6%)**	**Class 2: general discrimination (41.5%)**	**Class 3: education, income, weight, appearance and age discrimination (7.0%)**	**Class 4: compound discrimination (5.8%)**
Discrimination frequency	0.01	0.99	0.99	0.99
Gender	0.00	0.09	0.00	0.90
Race and ethnicity	0.00	0.04	0.05	0.34
Age	0.00	0.24	0.49	0.82
Education and income	0.00	0.12	0.67	0.38
Weight and appearance	0.00	0.16	0.62	0.49
Other discrimination^a^	0.00	0.04	0.24	0.39

a Other discrimination includes attributions to insurance/Medicaid status, ancestry/national origin, sexual orientation, and religion.

The likelihood-ratio difference test indicated that underlying LCA measurements differed significantly across racial and ethnic groups (∆G^2^ = 100.7, *df* = 56, *P < .*001). Thus, race/ethnicity–specific latent class models were estimated. Participants reporting other race and ethnicity were excluded from stratified LCA because of the small sample size. Fit indices and interpretability indicated a 4-class model as the best fit for each racial and ethnic group (Table 2).

### Race and ethnicity stratified models

Similar and different latent classes emerged in race and ethnicity stratified models (Table 3). Among all racial and ethnic groups, the “no discrimination” class was the largest (range, 45.6%–59.2%). The second largest class for each race and ethnicity (range, 31.0%–41.5%) was the “general discrimination” class, which experienced discrimination, although participants had a low probability of attributing discrimination to any particular characteristic. Only Hispanic participants in the general discrimination class had a moderate probability of attributing discrimination to race and ethnicity.

The 2 smaller classes of maternal discrimination in each race and ethnicity varied. Among Black participants, the third largest class (12.5%), “gender, race and ethnicity, and age discrimination,” experienced discrimination and had a high probability of attributing discrimination to gender, race and ethnicity, and age but a low probability of attributing discrimination to other characteristics. Participants in the fourth and smallest class (5.6%), “compound discrimination,” experienced discrimination and had a high probability of attributing discrimination to all characteristics.

Among Hispanic participants, the third largest class (6.1%), “other discrimination,” experienced discrimination and had a high probability of attributing discrimination to characteristics in the other discrimination category. The fourth and smallest class (3.6%), “compound discrimination,” experienced discrimination and had a high probability of attributing discrimination to all characteristics except age and weight and appearance, for which they had a moderate probability.

Among White participants, the third largest class (7.0%), “education, income, weight and appearance, and age discrimination,” experienced discrimination and had a high probability of attributing discrimination to education, income, weight and appearance, and age but a low probability of attributing discrimination to other characteristics. White participants in the fourth and smallest class (5.8%), “compound discrimination,” experienced discrimination and had a high probability of attributing discrimination to gender, age, and weight and appearance, as well as a moderate probability of attributing discrimination to other characteristics.

### Association with adverse perinatal health outcomes

Estimated outcome probability for each latent class and pairwise comparisons between each latent class are displayed in Table 4 and Table 5, respectively. Our focus is on results of the race and ethnicity–stratified models, as they were determined to best fit the data.

**Table 4:** Estimated Proportions of Adverse Perinatal Health Outcomes, by Latent Class, Centering and Racial Disparities Study

Item	No. (%)	BCH-estimated probabilities (95% CI)			
Overall (N = 2,286)		Class 1: no discrimination	Class 2: general discrimination	Class 3: education and income discrimination	Class 4: gender, race and ethnicity, and age discrimination
APHOs	731 (31.9)	0.32 (0.29–0.35)	0.33 (0.29–0.37)	0.31 (0.22–0.39)	0.31 (0.24–0.39)
PTB	205 (9.5)	0.09 (0.08–0.11)	0.12 (0.09–0.15)	0.04 (0.02–0.12)	0.09 (0.05–0.15)
LBW	186 (9.2)	0.08 (0.07–0.10)	0.09 (0.07–0.12)	0.10 (0.04–0.16)	0.11 (0.06–0.16)
SGA	353 (17.5)	0.17 (0.15–0.19)	0.17 (0.13–0.20)	0.21 (0.13–0.29)	0.18 (0.12–0.25)
NICU	38 (2.0)	0.02 (0.01–0.03)	0.02 (0.01–0.04)	0.01 (0.00–0.08)	0.02 (0.01–0.07)
Apgar <7	148 (6.7)	0.06 (0.05–0.08)	0.07 (0.05–0.09)	0.09 (0.04–0.14)	0.07 (0.03–0.12)
Preeclampsia	169 (7.4)	0.07 (0.06–0.09)	0.08 (0.06–0.10)	0.04 (0.01–0.10)	0.09 (0.06–0.15)
PPDS	154 (9.8)	0.21 (0.12–0.29)	0.06 (0.05–0.08)	0.12 (0.09–0.15)	0.10 (0.05–0.16)
**Black (n = 933)**		**Class 1: no discrimination**	**Class 2: general discrimination**	**Class 3: gender, race and ethnicity, and age discrimination**	**Class 4: compound discrimination**
APHOs	357 (38.3)	0.38 (0.33–0.42)	0.37 (0.31–0.43)	0.41 (0.30–0.51)	0.43 (0.25–0.62)
PTB	95 (10.8)	0.10 (0.07–0.13)	0.11 (0.07–0.15)	0.09 (0.02–0.15)	0.17 (0.03–0.31)
LBW	99 (12.4)	0.11 (0.08–0.14)	0.13 (0.09–0.18)	0.12 (0.06–0.22)	0.22 (0.09–0.42)
SGA	198 (24.8)	0.23 (0.19–0.28)	0.26 (0.20–0.31)	0.25 (0.17–0.37)	0.34 (0.18–0.55)
NICU	16 (2.1)	0.02 (0.01–0.04)	0.02 (0.01–0.05)	0.03 (0.01–0.11)	0.06 (0.01–0.26)
Apgar <7	75 (8.4)	0.08 (0.05–0.11)	0.09 (0.06–0.13)	0.11 (0.06–0.19)	0.06 (0.01–0.29)
Preeclampsia	77 (8.3)	0.08 (0.06–0.11)	0.08 (0.05–0.12)	0.13 (0.05–0.18)	0.06 (0.01–0.26)
PPDS	49 (7.7)	0.05 (0.03–0.08)	0.09 (0.06–0.15)	0.09 (0.04–0.20)	0.13 (0.04–0.38)
**Hispanic (n = 471)**		**Class 1: no discrimination**	**Class 2: general discrimination**	**Class 3: other discrimination**	**Class 4: compound discrimination**
APHOs	117 (24.8)	0.27 (0.22–0.33)	0.21 (0.15–0.29)	0.23 (0.09–0.46)	0.21 (0.06–0.54)
PTB	35 (7.8)	0.07 (0.05–0.11)	0.07 (0.04–0.14)	0.15 (0.05–0.39)	0.07 (0.01–0.47)
Apgar <7	22 (4.8)	0.05 (0.03–0.08)	0.02 (0.00–0.09)	0.14 (0.04–0.36)	0.15 (0.04–0.47)
**White (n = 853)**		**Class 1: no discrimination**	**Class 2: general discrimination**	**Class 3: education, income, weight, appearance, age discrimination**	**Class 4:** **compound discrimination**
APHOs	252 (29.5)	0.28 (0.25–0.34)	0.35 (0.29–0.40)	0.17 (0.06–0.41)	0.12 (0.04–0.30)
LBW	58 (7.7)	0.06 (0.04–0.09)	0.11 (0.08–0.15)	0.04 (0.00–0.39)	0.02 (0.00–0.33)
SGA	105 (14.0)	0.14 (0.11–0.18)	0.17 (0.13–0.22)	0.01 (0.00–0.97)	0.07 (0.02–0.26)
Apgar <7	50 (6.1)	0.06 (0.04–0.08)	0.07 (0.04–0.11)	0.08 (0.02–0.28)	0.02 (0.00–0.25)
Preeclampsia	62 (7.3)	0.07 (0.05–0.10)	0.08 (0.05–0.11)	0.07 (0.01–0.28)	0.07 (0.02–0.23)
PPDS	75 (9.3)	0.09 (0.07–0.14)	0.18 (0.13–0.25)	0.24 (0.09–0.49)	0.13 (0.04–0.34)

Abbreviations: APHOs, adverse perinatal health outcomes; BCH, Bolck, Croon and Hagenaars; LBW, low birthweight; NICU, neonatal intensive care unit; PPDS, postpartum depression symptoms; PTB, preterm birth; SGA, small for gestational age.

**Table 5:** Difference in Log Odds Estimations of Proportions of Outcomes, by Latent Class, Centering and Racial Disparities Study

Item	BCH-estimated difference in log odds (SE)					
	Class 2 vs class 1	Class 3 vs class 1	Class 4 vs class 1	Class 3 vs class 2	Class 4 vs class 2	Class 4 vs class 3
**Overall**						
APHOs	0.04 (0.11)	−0.05 (0.22)	0.98 (0.19)	0.91 (0.25)	0.93 (0.21)	1.02 (0.28)
PTB	0.29 (0.17)	−0.78 (0.55)	0.96 (0.32)	−0.06 (0.58)	0.68 (0.35)	1.74 (0.65)
LBW	0.16 (0.19)	0.21 (0.36)	1.29 (0.29)	1.05 (0.41)	1.14 (0.34)	1.09 (0.45)
SGA	−0.05 (0.15)	0.23 (0.26)	1.07 (0.24)	1.28 (0.31)	1.12 (0.27)	0.84 (0.35)
NICU	0.00 (0.40)	−0.35 (0.95)	1.07 (0.66)	0.65 (1.05)	1.07 (0.75)	1.42 (1.15)
Apgar <7	0.09 (0.16)	0.39 (1.18)	1.19 (0.34)	1.31 (0.43)	1.09 (0.06)	0.79 (0.19)
Preeclampsia	0.08 (0.19)	−0.74 (0.58)	1.27 (0.29)	0.18 (0.62)	1.19 (0.33)	2.01 (0.65)
PPDS	−1.36 (0.31)^a^	−0.63 (0.34)	0.18 (0.42)	1.73 (0.22)^a^	1.54 (0.34)	0.82 (0.36)
**Black**						
APHOs	0.99 (0.16)	0.14 (0.24)	1.24 (0.40)	0.15 (0.26)	0.25 (0.42)	1.11 (0.49)
PTB	1.09 (0.26)	−0.20 (0.45)	1.59 (0.53)	−0.29 (0.47)	0.51 (0.56)	1.79 (0.74)
LBW	1.23 (0.25)	0.07 (0.46)	1.83 (0.52)	−0.16 (0.43)	0.59 (0.54)	1.76 (0.70)
SGA	1.13 (0.19)	0.12 (0.29)	1.53 (0.46)	−0.01 (0.32)	0.39 (0.48)	1.41 (0.58)
NICU	1.04 (0.67)	0.59 (0.84)	2.37 (0.93)	0.55 (0.92)	1.33 (1.04)	1.77 (1.25)
Apgar <7	1.19 (0.28)	0.38 (0.39)	0.78 (0.94)	0.18 (0.42)	−0.41 (0.97)	0.41 (1.07)
Preeclampsia	0.91 (0.28)	0.22 (0.39)	0.55 (0.93)	0.31 (0.42)	−0.36 (0.96)	0.33 (1.06)
PPDS	1.74 (0.36)^b^	0.69 (0.53)	1.09 (0.75)	−0.05 (0.53)	0.35 (0.76)	1.39 (0.95)
**Hispanic**						
APHOs	−0.31 (0.27)	−0.23 (0.55)	0.69 (0.75)	0.08 (0.59)	1.00 (0.79)	0.92 (0.94)
PTB	−0.04 (0.45)	0.81 (0.69)	1.00 (1.25)	0.85 (0.79)	1.03 (1.31)	0.19 (1.43)
Apgar <7	−1.21 (1.05)	1.11 (0.71)	2.25 (0.86)	2.32 (1.29)	3.47 (1.37)	1.15 (1.08)
**White**						
APHOs	0.27 (0.17)	−0.65 (0.61)	−1.05 (0.58)	0.08 (0.65)	−0.31 (0.59)^b^	0.61 (0.83)
LBW	0.62 (0.30)^b^	−0.59 (1.48)	−1.24 (1.68)	−0.22 (1.54)	−0.86 (1.70)	0.36 (2.24)
SGA	0.25 (0.23)	−2.50 (3.92)	−0.70 (0.77)	−1.75 (3.97)	0.05 (0.79)	2.79 (4.02)
Apgar <7	0.22 (0.34)	0.34 (0.82)	−1.00 (1.41)	1.13 (0.91)	−0.22 (1.44)	−0.34 (1.61)
Preeclampsia	0.09 (0.31)	−0.04 (0.87)	0.04 (0.70)	0.88 (0.95)	0.95 (0.74)	1.08 (1.10)
PPDS	0.72 (0.29)^b^	1.10 (0.61)	0.35 (0.66)	1.38 (0.67)	0.63 (0.67)	0.25 (0.86)

Abbreviations: APHOs, adverse perinatal health outcomes; BCH, Bolck, Croon and Hagenaars; LBW, low birthweight; NICU, neonatal intensive care unit; PPDS, postpartum depression symptoms; PTB, preterm birth; SGA, small for gestational age.

a *P* < .01. *P* values determined by using Wald test.

b *P* < .05. *P* values determined by using Wald test.

Among Black participants, pairwise comparisons indicated that the expected probability of severe or moderate PPDS were significantly higher for the general discrimination class relative to the no discrimination class (9% vs 5%, *P = .*04). No other significant latent class differences were identified in the Black sample. Among Hispanic participants, pairwise comparisons did not show any significant between-class differences in outcomes that could be compared. Among White participants, pairwise comparisons indicated that the expected probability of severe or moderate PPDS for the general discrimination class was significantly higher than for the no discrimination class (18% vs 9%, *P = .*01). Additionality, the probability of LBW for the general discrimination class was significantly higher than for the no discrimination class (11% vs 6%, *P = .*04). Finally, among White participants, expected probability of composite APHO was significantly lower for the compound discrimination class than the general discrimination class (12% vs 35%, *P = .*02). No further significant differences in outcomes by class were observed in the White sample.

### Prenatal care assignment as an effect modifier

In analysis using maximum-probability assignment, prenatal care assignment was not found to significantly modify the relationship between discrimination subgroups and APHOs. However, among Black pregnant participants, prenatal care assignment significantly modified the relationship between discrimination subgroups and PPDS (β = 2.04, *P < .*05), such that individuals in the “gender, race and ethnicity, and age discrimination” class assigned to GPNC had 5.17 (95% CI, 1.56–17.11) times the odds of PPDS than those in the “No Discrimination” class, while individuals in the “gender, race and ethnicity, and age discrimination” class assigned to IPNC had 0.67 times the odds of PPDS relative to those in the no discrimination class.

## Discussion

We used an intersectionality framework to explore pregnant people’s varied and intersecting exposure to discrimination and its effect on birth outcomes. Discrimination varied significantly across race and ethnicity; therefore, models were estimated separately for each race and ethnicity. We identified 4 unique classes of self-reported discrimination. The largest 2 subgroups of discrimination in each race and ethnicity included participants who reported never experiencing discrimination (no discrimination) and participants who experienced discrimination but did not strongly attribute discrimination to any one characteristic (general discrimination). The smaller 2 subgroups were more varied, including one class with a high probability of attributing discrimination to a single or multiple characteristic and one class with a high or moderate probability of attributing discrimination to most or all characteristics. Discrimination subgroups identified are consistent with previous studies of intersectional discrimination, which have largely taken place among older adults (24–26) and in which similar classes of no/minimal discrimination, single/general attribution, several/multiple attributions, and high/all attributions were identified.

Further, we found pregnant people’s risk of developing some APHOs significantly differed by discrimination subgroup. Black and White participants experiencing general discrimination were found to be at an increased risk of PPDS relative to participants who did not experience discrimination. This finding is congruent with existing literature that demonstrates an association between discrimination and PPDS. Analysis of the Pregnancy Risk Assessment Monitoring System (PRAMS) postnatal survey suggests that respondents who report being upset by race-based discrimination in the prior year are more likely to identify as experiencing PPDS, with the strongest relationship seen for Black participants (27–29).

Contrary to previous studies, our analysis did not show a significant association between maternal discrimination and PPDS among Hispanic participants. This finding may reflect protective factors against the effect of discrimination in this community. Relative to other racial and ethnic groups, Hispanic participants had lower rates on all APHOs measured. A robust literature suggests that despite lower socioeconomic status, Hispanic people defy the socioeconomic gradient of health in demonstrating good health outcomes, a phenomenon known as the Hispanic Paradox (30). It is hypothesized that sociocultural norms and values such as social support and religiosity may buffer Hispanic people against health disparities (31). Studies find that the Hispanic Paradox deteriorates with increased time in the United States and among subsequent generations (32). Most Hispanic participants in our sample (65.6%) were born outside the US and may therefore have had less exposure to the social context inside the US.

Among White participants, those who experienced general discrimination also had a higher risk of delivering an LBW infant relative to participants who did not experience discrimination. Although this finding is consistent with extant literature supporting the association between self-reported discrimination and risk of APHOs, it being observed only among White participants was unanticipated, as the relationship has previously been seen to be most robust among Black pregnant people (8). White participants reported the highest rate of discrimination in our sample. We incorporated assessment of self-reported day-to-day discrimination based on multiple social identities; therefore, findings may be due to the high prevalence of discrimination based on social identities other than race and ethnicity among White participants. Relative to other racial and ethnic groups, White participants were most likely to be younger and to attribute their discrimination to age. Findings might also reflect differential measure interpretation across racial and ethnic groups. While many studies attest to the EDS’s strong psychometric properties, recent findings raise concerns about the instrument’s equivalence across diverse social groups (33). Interpretation of EDS questions may differ across racial and ethnic groups; White participants may be more likely to interpret the scale as asking about unfair treatment generally rather than specifically about social injustice (34).

An additional unexpected finding of our analysis was that White participants who experienced compound discrimination were less likely to experience an APHO relative to those who experienced general discrimination. This finding is the opposite relationship than would be predicted by an intersectionality framework and paired with other findings could suggest unique risks among the general discrimination subgroup. Alternatively, this finding may be an artifact of the compound discrimination subgroup’s small class size.

Although previous studies suggest that GPNC may reduce racial disparities in birth outcomes (35), GPNC was not found to buffer against the effects of discrimination class on APHOs in our study.

### Limitations

Our study has limitations. First, although our sample is relatively large, subanalyses by race and ethnicity further subdivide the sample, limiting power to detect differences in low prevalence outcomes. For this reason, discrimination subgroups representing discrimination attributed to one, many, or all characteristics may not have been significantly associated with greater risk of APHOs in our sample. Second, because the sample included largely Medicaid-eligible pregnant people with low medical risk from a single practice, findings may therefore not be generalizable to other populations. Moreover, our focus on medically low-risk pregnancies may have resulted in attenuated associations, particularly among Black people who might be expected to have worsened health at entry to prenatal care due to disadvantages across the life course. Finally, at this time the BCH distal outcome procedure in SAS is not equipped to accommodate covariates; therefore, our findings do not control for other potentially confounding factors. Although strategies exist that can accommodate covariates, the BCH approach has been found to be more accurate than these alternatives, considering uncertainty in class assignment (36,37). Analyses conducted using maximum-probability assignment should be interpreted with caution.

### Strengths

Our study also has several strengths. First, we applied a novel statistical approach, LCA, to explore experiences of discrimination during pregnancy. LCA moves beyond a single status analysis, providing a more comprehensive assessment of discrimination during pregnancy and its association with APHOs. The person-centered nature of LCA supports the application of an intersectional approach in which multiple social identities are jointly considered. Second, our study population was racially and ethnically diverse and was composed primarily of low-income participants. Finally, we had rigorous data collection, including variables from patient self-reported validated measures and through medical chart abstraction.

### Conclusion

This study enhances our understanding of discrimination in pregnancy and associated perinatal health outcomes which may inform strategies for perinatal health promotion. Findings highlight the importance of assessing and addressing discrimination as intersectional rather than unidimensional domains. Interventions adopting an intersectionality framework may be best suited to respond to the complex discrimination experiences that impact pregnant people and promote perinatal health. Screening for discrimination exposure as a significant risk factor for adverse perinatal health could be incorporated in prenatal care settings and a systematic surveillance system for discrimination exposure and perinatal outcomes implemented.

Our results align with existing evidence on perceived discrimination as a risk factor for APHOs. By incorporating an intersectionality framework, this study extends understanding of the variety and intersections of discrimination experienced by pregnant people, as well as the association with APHOs, particularly PPDS. Future research that uses a large and representative population-based data set is needed to further clarify subgroups most at risk, as well as factors that may moderate or mediate the deleterious effects of discrimination on perinatal health. Broader research suggests that these factors may include group identification, social support, resilience, and coping strategies (5). This work will be facilitated by the modification and validation of instruments to assess perceived discrimination for use across diverse social groups.
